# Inhibitory Effect of* Lactobacillus plantarum* FL75 and* Leuconostoc mesenteroides* FL14* against* Foodborne Pathogens in Artificially Contaminated Fermented Tomato Juices

**DOI:** 10.1155/2019/6937837

**Published:** 2019-02-26

**Authors:** Aïssé Bah, Helena Albano, Joana Bastos Barbosa, Imene Fhoula, Yosra Gharbi, Afef Najjari, Abdellatif Boudabous, Paula Teixeira, Hadda-Imene Ouzari

**Affiliations:** ^1^Laboratoire de Microorganismes et Biomolécules Actives (LR03ES03), Faculté des Sciences de Tunis, Université Tunis El Manar, Campus Universitaire, 2092, Tunis, Tunisia; ^2^Universidade Católica Portuguesa, CBQF-Centro de Biotecnologia e Química Fina-Laboratório Associado, Escola Superior de Biotecnologia, Rua Arquiteto Lobão Vital 172, 4200-374 Porto, Portugal

## Abstract

Tomatoes and tomato based-foods contain beneficial microorganisms and various organic acids that have important nutritional values for human. The objective of this study was to access the physiochemical properties of fermented tomatoes juices and to evaluate the competitiveness of lactic acid bacteria (LAB) against* Listeria monocytogenes*,* Listeria innocua*, and* Salmonella *spp., in artificially contaminated tomato juice. Microbial counting (LAB, fungi* Salmonella* spp., and* Listeria* spp.) was performed after fermentation and weekly during storage. Different organic acids (Lactic, succinic, and acetic) and ethanol were also monitored using HPLC method. Color parameters were also determined. The results showed an increase of lactic and acetic acid content, during fermentation and storage of juices inoculated with* Lactobacillus plantarum* and* Leuconostoc mesenteroides* at 25°C. Besides, citric acid and ethanol revealed higher content at the end of storage compared to that registered at 4°C. The pH from tomatoes juices decreased from an initial value of 4.5 to below 3.2. Alongside, foodborne pathogen population was significantly suppressed in tomatoes juices when the samples were coinoculated with LAB strains. Moreover, the inhibition of* Salmonella *species was faster compared to that of* Listeria*. After four weeks of storage at 4°C,* Lb. plantarum* and* Lc. mesenteroides* showed high survival rate, while pathogenic bacteria, yeasts, and molds cell numbers decreased drastically in all the contaminated vials. This work highlights the efficiency of* Lb. plantarum* and* Lc. mesenteroides* as potential starters for developing nutritious and safe fermented tomato juice products.

## 1. Introduction

Tomatoes are one of the major vegetables widely used throughout the world, either in fresh or in processed form, including canned, sun-dried tomatoes, juices, ketchup, mashed tomatoes, sauces, and soups [1]. Tomatoes juices are well recognized by their important nutritional values for human (low cholesterol, fiber and proteins, vitamins as well as *β*-carotene, potassium and lycopene, and high content of antioxidants) [2]. Fresh tomatoes, as well as grapes, lettuce, peaches, peppers, spinach, sprouts, are naturally colonized by large microbial populations. Pathogens (e.g.,* L. monocytogenes, E. coli, *and* Salmonella *spp.) can be part of these populations and may cause food poisoning when eaten as raw product [3]. However, the matrices of fresh tomato contain autochthonous beneficial microbes (e.g.,* Lb. plantarum, Lc. mesenteroides*) which may compete with pathogens and ensure extended shelf life [4, 5] Lactobacillus* plantarum* as well as other LAB were widely used in biopreservation of different food matrices [6]. Sugar content in juice is favorable for microbe proliferation and common foodborne pathogens, which may affect the quality of juice [7]. Fermentation by using LAB as starter carries out acidification, [8], which leads to the decrease of pH and production of lactic acid [9]. Besides, it improves the nutritional, rheological, and sensory properties of fruits [10]. Organic acid (lactic and acetic acid) produced may have an antimicrobial effect, which will therefore depend upon its pK value (dissociation constant) and the pH of the external medium [11]. The low pH of most fruits and vegetables reduces spoilage microbiota, besides, it favors the growth of LAB and fungi [12]. Microorganisms used as probiotics during fermentation of fruits and vegetables are recognized for their nutritional profile and for their health benefit [13]. However, heat treatment process (70°C, 10 min) destroys bacteria and inactivates enzymes [14]. Lactic acid bacteria affected the organic acid production during fermentation, from the metabolism of sugars [15]. Most strains of* Lactobacillus plantarum* have activity against fungal and spoilage microorganisms, in order to prevent adhesion, establishment, and invasion of enteropathogens such as* Salmonella* and* Listeria* [16]. The Food and Agriculture Organization (FAO) of the United Nations and the World Health Organization (WHO) reposed that if the* Listeria* count does not exceed 100 CFU/g at the time of consumption, the food is acceptable [17]. The preservation of vegetables juices by probiotic strains is an important technique for the elaboration of bioproducts and traditional food [18].

The objectives of this work were (i) to evaluate metabolites produced during fermentation and of storage of tomatoes juices, (ii) perform the microbiological analysis and viability of LAB used as starter, and (iii) test the antagonistic activity of LAB against foodborne pathogens in artificially contaminated vials.

## 2. Materials and Methods

### 2.1. Sampling and Preparation and Fermentation of Tomato Juice

Fresh tomatoes (*Lycopersicon esculentum*) were purchased from local markets (Portugal) and were taken to the laboratory for experimental analysis.

Tomato fruit was washed with tap water and the seeds as well as skin were removed. Then, the fruits were homogenized by conventional blender (Stomacher Mix® CC Click Clean®), for 8 min at room temperature. One hundred milliliters of tomato juices was distributed in 200 ml flask and 1 g of sucrose was added. The tomato juice was heated in the oven for 5 min at 70°C [4]. The starter strains (*Lb. plantarum *FL75 and* Lc. mesenteroides* FL14) used were isolated from spontaneous fermentation of tomatoes fruits (of Laboratory Microorganisms and Active Biomolecules “LMBA,” Tunisia). The protocol for processing and storage of tomatoes juices is described in Table 1.* Lactobacillus plantarum* FL75 and* Lc. mesenteroides* FL14 were mixed and inoculated as starter (4% v/v) in tomatoes juices leading to an initial cell number of 10^11^ and 10^9^ CFU/ml, respectively.

Also, juice samples were inoculated with pathogens including a mix of* S.* Typhimurium ATCC 14028*, S. *Enteriditis ATCC 13076,* S. *Braenderup H9812 [19] or a mix of* L. innocua *2030C (culture collection of Escola Superior de Biotecnologia ESB) and* L. monocytogenes* L7946 [20] at a final concentration of 10^6^ CFU/ml. Control samples were only inoculated with the mix of* Listeria* and* Salmonella* species, separately. The procedure was detailed in Table 1. Twelve samples were prepared and stored at 4°C and 25°C for 30 days as described by [4].

The inoculated juices and control samples were analyzed for growth and viability of strains after fermentation and at 7, 14, 21, and 28 days of storage. Also, physicochemical analyses and color determination were performed.

### 2.2. Microbiological Analyses

Viable cells were determined after each week during fermentation of tomatoes juices. One ml of tomato juice was homogenized in 9 ml of Ringer solution (Lab M). Juice preparation was serially diluted in Ringer solution and counts were done on different agar media: plate count agar (PCA) [21] was used for total counts of aerobic mesophilic (30°C for 48 h); Rose Bengal Chloramphenicol (RBC) was used for fungal and yeast (25°C for 5 days), Modified Semisolid Rappaport–Vassiliadis agar (MRSV) was used for* Salmonella *spp. and Oxford agar for* Listeria* spp. (37°C for 48 h) according to Stratakos et al. [22] and De Man-Rogosa-Sharpe agar (MRS) for lactic acid bacteria (30°C for 48 h–72 h).

### 2.3. Physicochemical Analyses

#### 2.3.1. Determination of pH, Carbohydrates, Organic Acids, Ascorbic Acid, Succinic Acid, and Ethanol

The level of pH was determined during each week of fermentation by using a Crison Micro pH 2002 pH-meter (Crison, Barcelona, Spain), equipped with an In Lab 427 puncture electrode (Mettler Toledo, Columbus, OH, USA).

After centrifugation of tomatoes juices (8877× g, 10 min, 4°C; Rotina 35R, Hettich, Germany), 2 ml of the obtained supernatants was filtered through a 0.20 *μ*m disposable syringe filter. The filtrate was analyzed by high-performance liquid chromatography (HPLC) equipped with a UV detector operated at 210 nm, using a Shim-pack SCR-101H column (7.9 mm × 30 cm). The concentrations of different compounds such as fructose and glucose, nonvolatile acids (citric, lactic, and ascorbic acids), acetic acid, and ethanol were determined. The analysis was performed at 30°C by using 100 mM perchloric acid as the eluent at a flow rate of 0.6 ml/min with a sample volume of 0.02 ml. Lactic and citric acids were identified by a comparison of the retention time of an authentic standard corresponding to each acid. The concentration was determined using a calibration curve obtained by different standard concentrations of each sample in the same conditions used for sample analysis. The assay was performed in duplicate according to Ferrari et al. and Barbosa et al. [23, 24].

#### 2.3.2. Color Measurements

The color measurements were performed to each sample each week during fermentation of tomatoes juices. The measurements were performed in the CIE (Commission Internationale de L'Eclairage) Lab color scale, using a Konica Minolta CR-300 Chroma Meter (Konica Minolta, Tokyo, Japan) colorimeter. The analysis consists in an evaluation of the color parameters L*∗*, a*∗*, and b*∗*. L*∗* value measures the lightness of the sample, ranging from 0 (black) to 100 (white), a*∗* varies between red (+a*∗*) and green (-a*∗*), and b*∗* varies between yellow (+b*∗*) and blue (-b*∗*) color space. Three color measurements were performed for each sample. The chroma value was calculated, which indicates the color intensity and saturation (Chroma= (a*∗*2 + b*∗*2) ^1/2) and the hue angle, which measures the highlights, midtones, and shadows (Hue angle=tan-1(b*∗*/a*∗*) [25].

## 3. Results and Discussion

### 3.1. Microbiological Analysis

**Figure 1:**
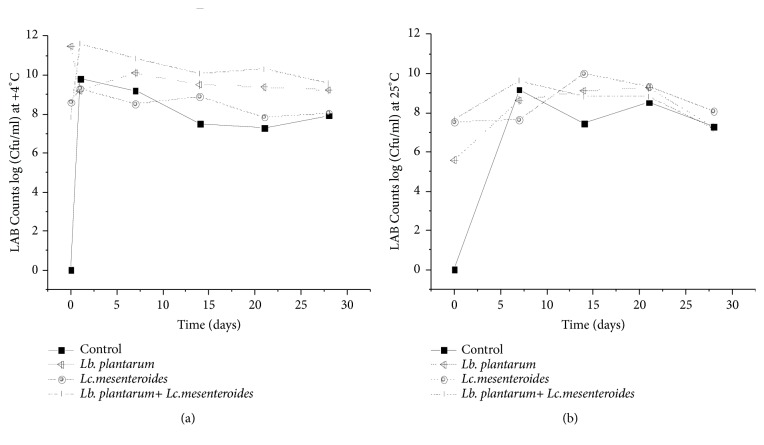
The growth of LAB in tomatoes juices after 24 h of fermentation and during storage at 4°C (a) and 25°C (b).

Cell counts of LAB ranged between 10^8^ and 10^11^ CFU/ml during the fermentation (Figure 1). Moreover, LAB counts of tomatoes juices inoculated with strains of* Lc. mesenteroides *and* Lb. plantarum* remain high during the first weeks until 21 days of storage. The result was similar to that obtained by Mousavi et al. [26]. Cell viability obtained at the beginning of fermentation in the tomatoes juice inoculated with LAB was higher compared to the control sample.

**Figure 2:**
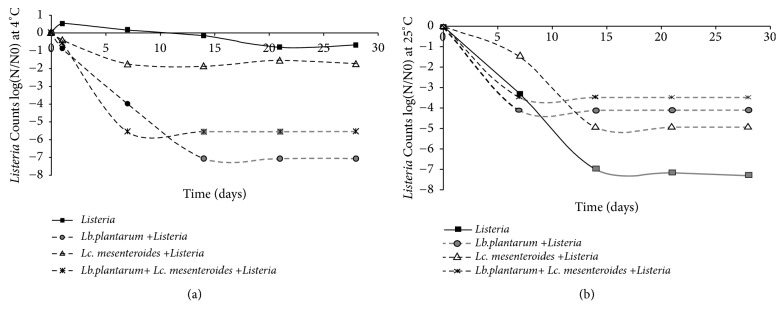
Logarithmic reduction of* Listeria* species by LAB in tomatoes juices after 24 h of fermentation and during storage at 4°C (a) and 25°C (b). The control used corresponds to* Listeria*(black square with solid line),* Lb.plantarum*+* Listeria*(gray circle with dashed line),* Lc. mesenteroides*+* Listeria*(empty triangle with dashed line), and* Lb.plantarum*+* Lc. mesenteroides* +*Listeria*(asterisk with dashed line). The gray lines mean that the isolate was reduced to values below the detection limit of the enumeration technique.

Fast growth of lactic acid bacteria in tomatoes juices showed to be advantageous, because there was production of organic acid, resulting in rapid fermentation periods. As indicated by Pereira et al. [27], tomato juice inoculated with* Lb. plantarum* and* Lc. mesenteroides* has showed high production of organic acids compared to the control samples in both conditions. Lactic acid and low pH in juices influence the viability of LAB at the end of storage in fermented tomato juice samples at 4 and 25°C. This result confirms the metabolism effect of the* Lb. plantarum* and* Lc. mesenteroides *used as starter for tomatoes juices acidification. Higher pH values in the control samples could be due to the occurrence of autochthonous lactobacilli with low acidifying effect power. In addition, high pH value of the control samples could be due to the superficial development of molds which lead to the loss of acidification and deaminase activities as also reported by Merchesini et al. or Sunesen and Stahnke [28, 29]. The number of yeasts and molds was increased gradually from day 14 of fermentation for both inoculated and control tomato juices. However, in the juices inoculated with* Lb. plantarum* FL75 and* Lc. Mesenteroides *FL14, the cell density of spoilage yeasts and molds was markedly lower than that found frequently in samples subjected to spontaneous fermentation (control), indicating the antifungal action of LAB used as starter. These findings are similar to other studies conducted either for fermented foods [30] or for food preservation [31, 32]. Antilisterial activity by LAB (*Lb. plantarum* and* Lc. mesenteroides*) coinoculated in tomatoes juices was detected by diminishing the growth rate of* Listeria* at the end of fermentation and its total inhibition after one week of storage conditions (Figure 2). This behavior was also observed when* Lb. plantarum* was inoculated alone, with the difference that total inhibition of* Listeria* was obtained after two weeks of storage at 4°C. However, when tomatoes juices were inoculated with* Lc. mesenteroides*, total inhibition was observed only for storage conditions of 25°C, after two weeks. Such difference in viability upon storage temperature may be due to the reduced growth rate and secondary metabolites release at 4°C. Besides, total inhibition of* Listeria* was not observed for the control samples. It was only reduced to 2.1 CFU/ml at 25°C. The obtained results highlight the importance of use and selection of starters and the effectiveness of* Lb. plantarum* compared to* Lc. mesenteroides* in inhibiting* Listeria* population. As it was previously reported by Alves et al. and Albano et al. [33, 34], antilisterial activity was due to the effect of LAB, which showed high viability rate after fermentation and during storage (Figure 2).

**Figure 3:**
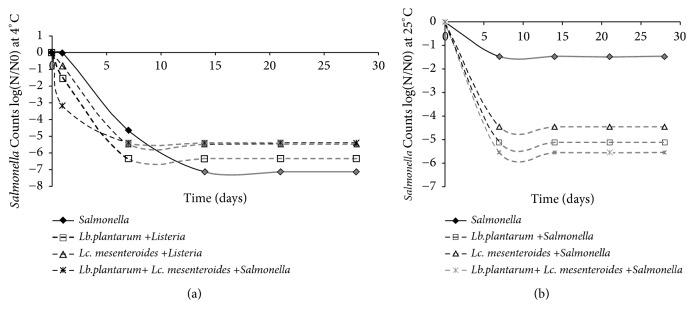
Logarithmic reduction of* Salmonella *species by LAB in tomatoes juices after 24 h of fermentation and during storage at 4°C (a) and 25°C (b). The control used corresponds to* Salmonella*(black diamond with solid line),* Lb.plantarum*+* Salmonella*(empty square with dashed line),* Lc. mesenteroides*+* Salmonella*(empty triangle with dashed line), and* Lb. plantarum*+* Lc. mesenteroides* +* Salmonella*(asterisk with dashed line). The gray lines mean that the isolate was reduced to values below the detection limit of the enumeration technique.

Similarly, monitoring of* Salmonella *viability in contaminated tomato juice revealed that when LAB count was increased, the cell counts of* Salmonella* were reduced after storage at 4 and 25°C for 28 days. In control samples,* Salmonella* counts were also decreased progressively during storage at 4°C and slightly differ from which observed in samples kept at 25°C (Figure 3), suggesting the involvement of various antimicrobial compounds (acetic and lactic acid, hydrogen peroxide (H_2_O_2_), and bacteriocin). This result was in accordance with those obtained by Li et al. [35], showing an antagonistic effect by LAB against* Salmonella*. These findings confirm the observations obtained by Fazeli et al.; Brillet et al.; and Budde et al. [36–38], which suggested that application of selected LAB as starter cultures allowed fast growth rate in the fermented products at different temperatures. LAB counts increased rapidly at the beginning of fermentation, allowing the pH decrease, due to the metabolic activity of LAB which inhibit pathogens.

### 3.2. Physicochemical Analyses

**Figure 4:**
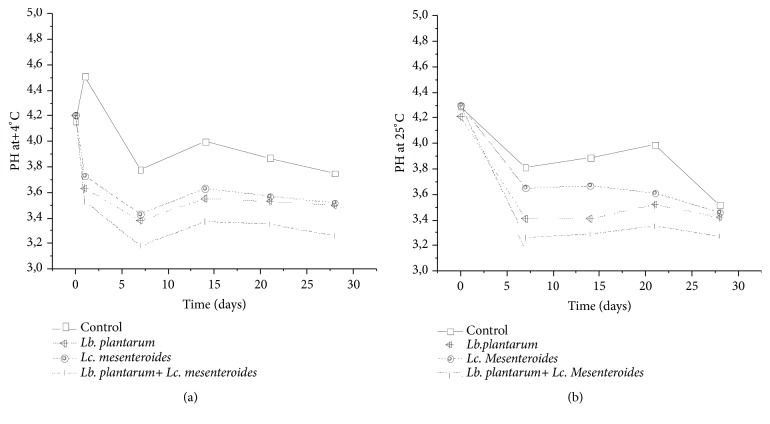
pH variation monitoring in tomatoes juices after 24 h of fermentation and during storage at 4°C (a) and 25°C (b).

The results showed that the evolution of pH in tomatoes juices was similar in both storage conditions at 4°C and 25°C (Figure 4). The pH was decreased progressively from initial value 4.2 to about 3.6, after 7 days and 3.4 after 28 days, while the control sample revealed a slightly higher pH compared to the inoculated samples. The pH of tomatoes juices inoculated with* Lb. plantarum* FL75 varied from 4.2 to 3.38 after 14 days of storage at 4°C, with a slight increase until 3.55 after 21 days. This finding is in agreement with similar studies reported by Georgieva et al. [39]. High pH values in the control samples and all samples inoculated after 14 days of storage could be due to the occurrence of molds. Moreover, the coinoculated samples with* Lb. plantarum* FL75 and* Lc. mesenteroides* FL14 showed a lower pH in all the analyzed samples. Accumulation of organic acids confirmed the strong acidifying activity of LAB used as starter, which is in concordance with those obtained by Di Cagno et al. [10].

**Figure 5:**
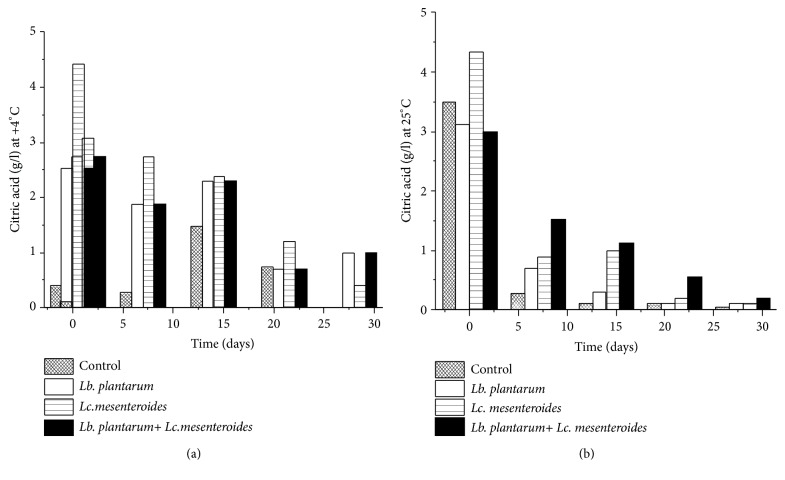
Citric acid quantification in tomatoes juices after 24 h of fermentation and during storage at 4°C (a) and 25°C (b).

**Figure 6:**
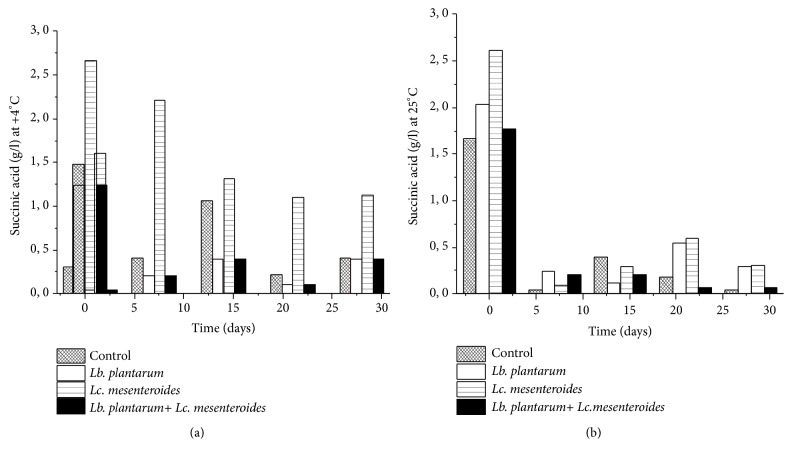
Evolution of Succinic acid amount after 24 h of fermentation and during storage at 4°C (a) and 25°C (b).

The concentrations of citric acid, L-ascorbic acid, and succinic acid were decreased in all tomatoes juices subjected to spontaneous fermentation and starter-fermented tomatoes juice (Figures 5 and 6). The L-ascorbic acid content diminished from 176 mg/l to 46.43 mg/l. Besides succinic acid content ranged to 2.7g/l and to 1.12 g/l in tomatoes juices inoculated with* Lc. mesenteroides* FL14 during storage at 4°C, which is in concordance with Hernández et al. [40].

The degradation of ascorbic, succinic, and citric acid was greater rapidly during storage at 25°C compared to 4°C (Figures 5 and 6). This is due to the use of citric, succinic, and ascorbic acid by LAB as energy source during storage.

**Figure 7:**
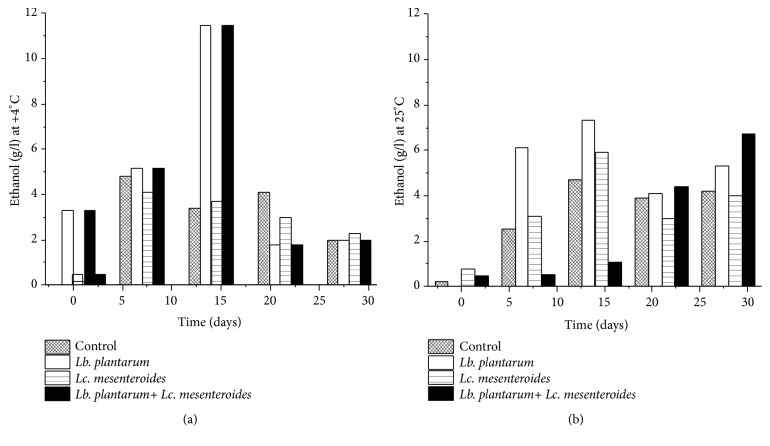
Production of bioethanol in tomatoes juices after 24 h of fermentation and during storage at 4°C (a) and 25°C (b).

However, the concentrations of ethanol were increased with a maximum at day 14 in both storage temperatures followed by a slight decrease (Figure 7). The concentrations of ethanol were increased in tomatoes juices inoculated with* Lb. plantarum *FL75 and* Lc. mesenteroides* FL14 at the end during storage of 25°C, which suggests that the inoculated LAB were totally responsible for ethanol production [41, 42]. Heating of tomatoes juices before starter cultures inoculation decreases the load of foodborne pathogenic bacteria and promotes the growth and proliferation of lactic acid bacteria during fermentation and storage, leading to the production of healthy and nutritional tomatoes juices, which verify the properties probiotic beverages [43]. The growth of LAB depends on the substrate such as glucose (very good carbon and energy source) and even on the fermentation time, which explained the reduction of carbohydrates (glucose and fructose) in tomatoes juices stored at 4°C and 25°C. This result is in agreement with those of Reddy et al. [44], obtained for mango juice fermentation.

**Figure 8:**
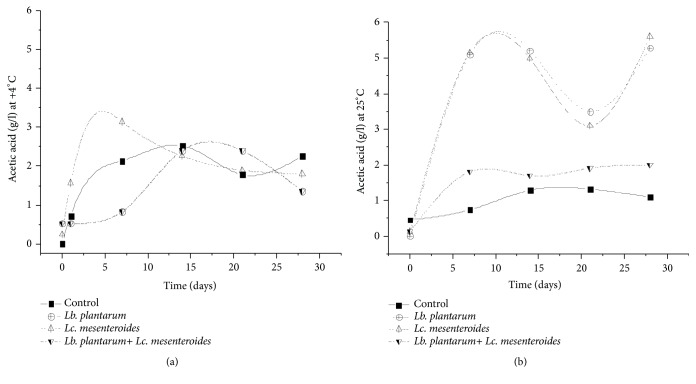
Acetic acid content in tomatoes juices after 24 h of fermentation and during storage at 4°C (a) and 25°C (b).

**Figure 9:**
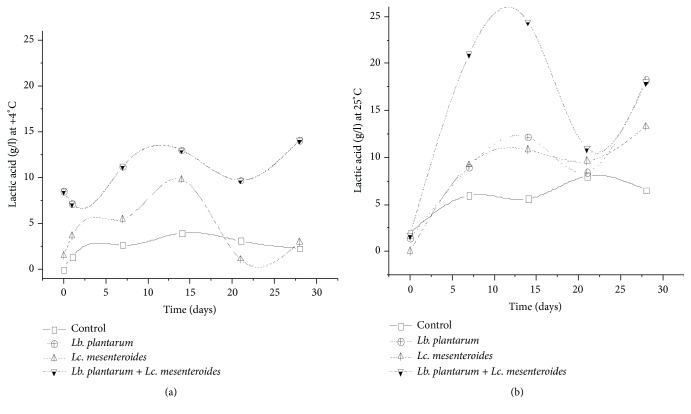
Concentration of lactic acid in tomatoes juices after 24 h of fermentation and during storage at 4°C (a) and 25°C (b).

Besides, organics acids were determined to evaluate their content variation upon LAB starter use in the tomatoes juices and control sample during fermentation and storage. Organic acid such as lactic and acetic acids has shown high amount in tomatoes juices inoculated with LAB starter compared to the control sample (Figure 8), which can affect the flavor of the final product due to the activity of lipases. However, higher content was noticed for samples inoculated with* Lb. plantarum* compared to those inoculated with* Lc. mesenteroides *(Figure 9). Higher amount of acids was also registered for juice samples inoculated with LAB and stored at 25°C than juices stored at 4°C. These results are in agreement with Kohajdova et al. and Perez et al. [21, 45], which reported the production of organic acids by* Lb. plantarum* in vegetables juices.

Fruit acidity and sweetness are among the major factors determining the quality of tomatoes juices, by decreasing the pH during fermentation and storage, which correlated with the high quantity of organic acids [46].

As indicated by Essid et al. [30], the acidifying activities of* Lb. plantarum in vivo* were demonstrated by inhibition of spoilage microorganism of* Listeria* and* Salmonella* in the inoculated tomatoes juices compared to the control samples. The antimicrobial activity is translated by the undissociated form of the acid which can cross the microbial membrane and inhibit pathogenic bacteria such as* Salmonella*,* Listeria*, and the spoilage molds. According to the Henderson-Hasselbalch equation [47] the antimicrobial activity is effective when the amounts of acetic acid and lactic acid have reached 0-22 mg/g and 1-68 mg/g, respectively. Strong accumulation of lactic and acetic acids in tomatoes juices suggests antimicrobial activity against* Salmonella*,* Listeria*, and spoilage molds,* in vivo*. Moreover, in accordance with Jankuloski et al. [48], pathogenic bacteria and the spoilage molds were decreased after 14 days of storage at 4°C and 25°C, due of the effect of acetic and lactic acids in all tomatoes juices.

**Figure 10:**
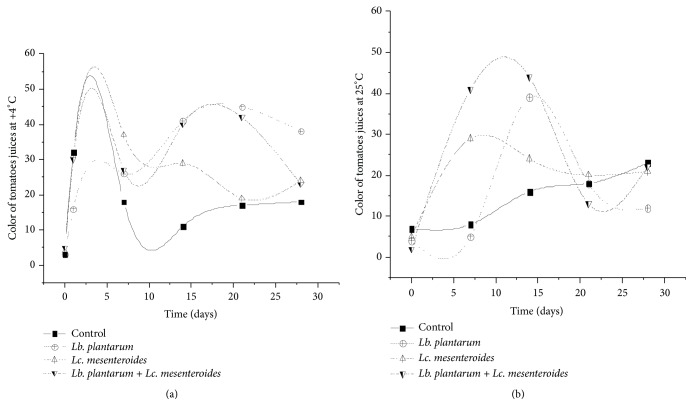
Color component of tomatoes juices.

Red color of tomatoes juices is related to the concentration of its pigments (carotenoids and lycopen) and is one of the organoleptic properties often evaluated. Ours results indicated intense color development in tomato juices inoculated by LAB compared to the control samples (Figure 10). Moreover, juices samples inoculated only with* Lb. plantarum* or the mixed of* Lb. plantarum* and* Lc. mesenteroides* showed higher color intensity than those inoculated only by* Lc. mesenteroides*, particularly after ten days of storage. These results are in agreement with those of Gould et al. [49], reporting the variation of color during storage of tomato juice.

## 4. Conclusion

The results of this study highlight the effectiveness of LAB starter in inhibiting foodborne pathogens of* Listeria* spp. and* Salmonella* spp. species during fermentation and storage of fermented tomatoes juices.* Lb. plantarum* associated or not with* Lc. mesenteroides* have shown high viability in the tomato matrix and has exhibited high level of organic acids, ethanol, and color development. The selected LAB starter has proven efficiency in the development and biopreservation of dietetical and safe functional food with important shelf life at both room and refrigerated temperatures.

## Figures and Tables

**Table 1:** Different treatment of tomatoes juices.

Samples	Code
1. Non inoculated juice	Control
2. Juices inoculated with *Lb. plantarum*	*Lb. plantarum*
3. Juices inoculated with *Lc. Mesenteroides*	*Lc. mesenteroides*
4. Juices inoculated with *Lb. plantarum* and *Lc. mesenteroides*	*Lb. plantarum+ Lc. mesenteroides*
5. Juices inoculated with Mix of *Salmonella* species and *Lb. plantarum*	*Salmonella*+ *Lb. plantarum*
6. Juices inoculated with *Salmonella* species and *Lc. mesenteroides*	*Salmonella* + *Lc. mesenteroides*
7. Juices inoculated with *Salmonella* species, *Lb. plantarum* and *Lc. Mesenteroides*	*Salmonella*+ *Lb. plantarum*+ *Lc. mesenteroides*
8. Juices inoculated with Mix of *Salmonella* species	*Salmonella*
9. Juices inoculated with Mix of *Listeria* species	*Listeria*
10. Juices inoculated with *Listeria* species and *Lb. plantarum*	*Listeria*+ *Lb. plantarum*
11. Juices inoculated with *Listeria* species and *Le. mesenteroides*	*Listeria*+ *Lc. mesenteroides*
12. Juices inoculated with* Listeria* species, *Lb. plantarum* and *Lc. mesenteroides*	*Listeria*+ *Lb. plantarum*+ *Lc. mesenteroides*

## Data Availability

The data used to support the findings of this study are available from the corresponding author upon request. The nucleotide sequences data used to support the findings of this study are publicly available in the GenBank repository at National Center for Biotechnology Information NCBI (https://www.ncbi.nlm.nih.gov/genbank/), Lactobacillus plantarum FL75 MH037134; Leuconostoc mesenteroides FL14 MH037132.

## Supplementary Materials

Supplementary Materials The result of Biolog phenotypic microarray analysis data used to support the findings of this study are included within the supplementary information file.
