# Supplementary material for: Inhibitory Effect of Lactobacillus plantarum FL75 and Leuconostoc mesenteroides FL14 against Foodborne Pathogens in Artificially Contaminated Fermented Tomato Juices
Source: Biomed Res Int. 2019 Feb 26;2019:6937837. doi: 10.1155/2019/6937837 (PMC6413396; doi:10.1155/2019/6937837)
Supplement: Supplementary Materials — The result of Biolog phenotypic microarray analysis data used to support the findings of this study are included within the supplementary information file. [file 6937837.f1.pdf]

Supplemental materials

Phenotype Microarrays

The growth on different carbon sources (93) of two *Weissella* strains was assessed using Phenotype Microarray (PM) Technology (Biolog, Hayward, CA). Bacterial cells from a single colony, grown on BHI agar for 48 h were suspended in the specific Biolog medium (adjusted to 65% of transmittance) and used to inoculate the phenotype microarray 96-well plates (PM1 and PM2), according to the manufacturer's instructions. PM plates were incubated for 72 h at 37°C. Data from a single experiment were analyzed with Omnilog-PM software. For each carbon source, the metabolic activity was measured quantitatively based on the area under curve. The two independent replicates of each PM plate showed the same results.

Supplementary Table S1: Positive reaction for carbohydrate utilization by two *Weissella halotolerans* strains using Biolog phenotypic microarray.

Substrats	*W. halotolerans* F99	*W. halotolerans* FAS24
**L-Arabinose**	+++	-
**N-Acetyl-D- Glucosamine**	+++	++
**D-Galactose**	+++	-
**D-Trehalose**	+++	-
**D-Mannose**	+++	+
**Dulcitol**	-	+++
**Glycerol**	-	+++
**D-Gluconic Acid**	+++	+++
**D-Xylose**	++	-
**D-Mannitol**	+++	-
**D-Ribose**	+++	++
**Tween 20**	+++	-
**D-Fructose**	+++	-
**Alpha-D-Glucose**	+++	-
**Maltose**	+++	-
**Thymidine**	+++	-
**Tween 40**	+++	-
**Alpha-D-Lactose**	-	-
**Uridine**	+++	++
**Tween 80**	+++	-
**Maltotriose**	-	++
**Adenosine**	++	-
**D-Cellobiose**	++	-
**Inosine**	++	+
**N-Acetyl-beta-D- Mannosamine**	-	++
**L-Lyxose**	-	+++
**2-Aminoethanol**	+	++
**Alpha-Cyclodextrin**	+	-
**Beta-Cyclodextrin**	+	-
**Gama-Cyclodextrin**	+	-
**Dextrin**	+	++
**N-Acetyl-D- Galactosamine**	-	-
**D-Arabinose**	+++	+
**Arbutin**	+	+++
**2-Deoxy-D- Ribose**	+++	+++
**Gentiobiose**	+++	-
**L-Glucose**	-	-
**a-Methyl-D- Glucoside**	+++	-

(-), absence of activity; (+), weak activity; (++), moderate activity; (+++), strong activity.
